# Transition to Parenthood and HIV Infection in Rural Zimbabwe

**DOI:** 10.1371/journal.pone.0163730

**Published:** 2016-09-29

**Authors:** Emanuele Del Fava, Raffaella Piccarreta, Simon Gregson, Alessia Melegaro

**Affiliations:** 1 Carlo F. Dondena Centre for Research on Social Dynamics and Public Policy, Bocconi University, Milan, Italy; 2 Department of Decision Science, Bocconi University, Milan, Italy; 3 Department of Infectious Disease Epidemiology, Imperial College London, London, United Kingdom; 4 Biomedical Research and Training Institute, Harare, Zimbabwe; 5 Department of Policy Analysis and Public Management, Bocconi University, Milan, Italy; Tulane University School of Public Health and Tropical Medicine, UNITED STATES

## Abstract

**Background:**

The relationship between the risk of acquiring human immunodeficiency virus (HIV) infection and people’s choices about life course events describing the transition to parenthood–sexual debut, union (in the form of marriage, cohabitation, or long-term relationship), and parenthood–is still unclear. A crucial role in shaping this relationship may be played by the sequence of these events and by their timing. This suggests the opportunity to focus on the life courses in their entirety rather than on the specific events, thus adopting a holistic approach that regards each individual’s life course trajectory as a whole.

**Methods:**

We summarise the individual life courses describing the transition to parenthood using ordered sequences of the three considered events. We aim to (*i*) investigate the association between the sequences and HIV infection, and (*ii*) understand how these sequences interact with known mechanisms for HIV transmission, such as the length of sexual exposure and the experience of non-regular sexual partnerships. For this purpose, we use data from a general population cohort study run in Manicaland (Zimbabwe), a Sub-Saharan African area characterised by high HIV prevalence.

**Results:**

For both genders, individuals who experienced either premarital or delayed childbearing have higher HIV risk compared to individuals following more standard transitions. This can be explained by the interplay of the sequences with known HIV proximate determinants, e.g., a longer exposure to sexual activity and higher rates of premarital sex. Moreover, we found that people in the younger birth cohorts experience more normative and safer sequences.

**Conclusions:**

The shift of younger generations towards more normative transitions to parenthood is a sign of behaviour change that might have contributed to the observed reduction in HIV prevalence in the area. On the other hand, for people with less normative transitions, targeted strategies are essential for HIV prevention.

## Introduction

The estimated number of people living with human immunodeficiency virus (HIV) at a global scale has consistently increased from 33.3 million in 2010 to 36.7 million (47% of which were women) in 2015 [1]. At the same time, there has been a steady decline in the annual number of new HIV infections (from 2.2 million in 2010 to 2.1 million in 2015), and of AIDS-related deaths (from 1.5 million in 2010 to 1.1 million in 2015), mostly due to changes in sexual behaviours and to the rising number of people receiving antiretroviral therapy (from 7.5 million in 2010 to 17 million in 2015) [1]. The world region that is most affected by HIV is still Sub-Saharan Africa (SSA), accounting for the 69.5% of the total number of people living with HIV in 2015 [1]. The HIV prevalence in this region in 2013 was around 4.7% among people aged 15–49, with peaks in Southern African countries, from Swaziland (27.4%) to Zimbabwe (15%) [2].

Also, a great variation in the size of the HIV prevalence among adults has been registered within SSA countries [3,4]. Among the factors proposed to explain this heterogeneity are those related to individual sexual behaviours–such as multiple sexual partnerships outside the union (i.e., marriage, cohabitation, and long-term relationship), lack of condom use, and male circumcision–and to other sexually transmitted infections [4]. Nonetheless, also major life course decisions taken during adolescence and early adulthood and driving the transition to parenthood, such as entering a long-term relationship or having a child, may be considered as relevant factors in the explanation of the observed differences of HIV epidemics within countries [5–7]. Even though these demographic events are usually considered only as underlying determinants of HIV infection [8], they actually shape individuals’ exposure to either risky or protective behaviours over time, acting as enabling circumstances for HIV transmission [9].

A number of studies have focused on the relationship between the demographic events leading to parenthood, namely, sexual debut, union and parenthood–and the adoption of risky sexual behaviours that increase the risk of HIV infection [10–18]. Even so, results on this matter are not unidirectional. Indeed, the final outcome of each specific personal experience seems to be related to its timing with respect to the other family-related decisions. For instance, early union (indicating both formal marriage and consensual union) is associated with a higher HIV risk for young inexperienced women, who often get married with older men and may be greatly exposed to unprotected sex [12,19]. On the other hand, the postponement of union turned out to be associated with a higher HIV risk, as a large interval between sexual debut and union may leave one exposed to higher rates of sexual partner change [13,20].

In fields like demography and ethnography, there is a large body of research on life course events experienced in the transition to adulthood and parenthood [21–23]. A number of studies focus on Sub-Saharan Africa [24–26], and on the juxtaposition between “ordered” or socially “normative” life course trajectories and “disordered” ones [22,27]. Considering the transition to parenthood, the life course perspective considers personal decisions on, e.g., union, as necessarily connected to decisions on sexual debut and/or childbearing. For instance, a woman may enter a stable union and have her sexual debut within it, or rather experience sexual debut and pregnancy outside of a relationship, or never have children. In other words, the idea behind the so-called *holistic* life course approach is that the life course trajectories summarise all the preferences, decisions and incidents that might occur during a person’s life [28].

Also, there is an increasing support towards the idea that health is the result of a continuous process that develops over an individual’s lifetime [29–32]. This, combined with the strict interconnection between life course events, makes it challenging to investigate the association between the possible role of the timing and/or of the ordering of the experienced life course events on the final health outcome. For example, does union have the same effect on HIV, irrespective of whether it precedes or follows childbirth? As a matter of fact, we found that, despite the vast literature on life course analysis and life course epidemiology, very little work has been done on the relationship between life course trajectories experienced during the transition to parenthood and HIV infection [5,7]. We think instead that this topic deserves attention. For example, observing that a certain life course trajectory is associated with a higher HIV risk suggests that individuals exhibiting such a trajectory took decisions and underwent experiences in their life, in terms of timing of events and sexual risk behaviours that led them to incur in a higher chance of being infected.

Along these lines, our study focuses on the life course trajectories describing the transition to parenthood, and specifically on the ordering and the timing of the involved focal events (sexual debut, union, and parenthood itself). Our goal is to understand whether such life course trajectories are associated with HIV infection through known proximate mechanisms, such as a longer sexual exposure combined with early sexual debut [10,11,16,18], and the experience of non-regular sexual relationships [33–35].

To investigate this association, we use data from a general population cohort study carried out in Manicaland [36], a rural province of Zimbabwe, a country in Southern Africa that is also one of the poorest and most HIV-affected countries in the world. Even so, Zimbabwe experienced a strong decline in the HIV incidence, passing from 130,000 new HIV infections in 2001 (HIV prevalence at 24.3%) to 69,000 in 2012 (HIV prevalence at 14.7%) [37]. This has been mostly related to changes in sexual behaviour and, in particular, to the decrease of multiple sexual partnerships [38].

For each individual, we summarise the transition to parenthood using event sequences built based on the three focal events (sexual debut, first union, and first child). We propose a new method to account for the peculiar characteristics of the problem and the data at hand. Specifically, we develop a procedure to deal with censored sequences (when the individual has not experienced all the three events by the time of the last interview) and with delayed events (when a person experiences a certain event much later with respect to his peers). Using logistic regression, we then study the association between the sequences and the probability of testing HIV-positive, conditioned to gender. We finally analyse if and to what extent such association can be explained by sexual behavioural factors recognised as relevant in the literature.

## Materials and Methods

### Study setting

Our data arise from the Manicaland HIV Prevention Study, a general population cohort study carried out between 1998 and 2011, with participants recruited in five rounds: 1998 to 2000 (Round 1), 2001 to 2003 (Round 2), 2003 to 2005 (Round 3), 2006 to 2008 (Round 4), and 2000 to 2011 (Round 5) [36]. The study was conducted in 12 sites, differing in terms of socioeconomic level: two small townships, four forestry/tea/coffee estates, two roadside trading settlements, and four subsistence farming areas. In the area, only starting with the years 2000s, services aimed at HIV prevention and treatment were implemented. In particular, prevention mother-to-child transmission (PMTCT) services started around 2003. Antiretroviral therapy (ART) services started around 2005, but they were really scaled-up from 2009 due to the economic crisis that hit the country between 2006 and the early 2009.

### Study design

At each study round and in each selected study site, men and women aged 15–54 years were recruited based on a household census, and were asked to answer a structured interview, aimed at retrospectively collecting socio-demographic and health-related information. The interview included a number of questions related to sexual behaviour that were asked using a blend of face-to-face interview (FTFI) and self-completion methods, namely, the *Informal Confidential Voting Interview* (ICVI), so as to reduce social desirability bias [39]. Even though ICVI’s efficacy seems to be highly dependent on temporal [40] and local [41] circumstances, it was proved to be more effective than FTFI for the reporting of the number of sexual partners [39].

At the end of the interview, respondents were given the opportunity to ask questions. Quite often, they would ask for the correct answers to survey questions on HIV awareness. Moreover, they were asked to take an anonymous HIV serological test. Written informed consent was sought, blood spots were collected on filter paper from a finger prick, and dried blood spots were analysed using a highly sensitive and specific antibody dipstick assay [42]. Being the test anonymous, survey respondents were not provided with their results. However, independently from the study, they were offered free HIV voluntary counselling and testing (VCT). The service was made available at a mobile clinic that was present within the study site at the time the survey was being conducted [42]. Participants who opted for VCT could do so after completing the survey questionnaire. Prior ethical approval was granted by the Medical Research Council of Zimbabwe, Harare-based Biomedical Research and Training Institute’s Institutional Review Board, and the Imperial College London Research Ethics Committee. The collected data were anonymised before performing any analysis.

The overall response rates in the study varied between rounds from 73% to 87%, with response rates being similar in peri-urban and rural areas. Overall follow-up rates between rounds varied from 48% (over 3 years) to 61% (over 2 years). Excluding deaths and out-migrants (who were not eligible for follow-up), these rates increased to 77% (3 years) and to 97% (2 years). Of course, quite a few new people migrated into study areas, which, to some extent, might have compensated for those leaving.

All the individuals who ever participated in the study were initially considered as eligible for inclusion in our sample. Additional criteria for inclusion in our analysis were (i) being sexually experienced at the time of the last interview; (ii) having provided information on HIV testing history (irrespective of whether or not the respondent had been previously tested or not); (iii) having provided dried blood spots for HIV testing at least once during the study, and having a clear test result. The total number of eligible respondents fulfilling our inclusion criteria was 27,073, of which 15,949 women and 11,124 men (S1 Table).

Retrospective self-reported data provided by respondents on their age (in years) at the events of interest were used in the current analysis. As already noted for the Manicaland Study and for other similar studies on SSA countries, a high proportion of unreliable reports of both age at first sex and age at first union was found among individuals attending multiple surveys [43,44]. The same problem was found for the age at first child. Inaccurate reporting of ages at events might depend on several factors. Questions on sexual debut might be at risk of social desirability bias, with women tending to under-report sexual activity, and men over-reporting it [45–48]. Among older people, recall bias resulting in inaccurate reporting across rounds may be most common due to the longer gap between the time when the events actually occurred and the date of the interview [44]. In those cases when it was possible to suitably address inconsistencies (for example, when the inconsistency was found only in one wave), individuals were kept in the sample. Otherwise, they were excluded. Nevertheless, this action should have not resulted in artificial generation or suppression of trends [43,44].

After the removal of inconsistent data, our sample was made up of 11,647, of which 6,829 women and 4,818 men, with their information updated at the last available interview (S1 Table).

### HIV testing

The HIV test taken as part of the study, based on the dipstick assay applied to the respondents’ dried blood spots, was used for research purposes. Being the testing procedure anonymous, and not deemed accurate enough for diagnostic purposes, survey respondents were not provided with their results. Therefore, a respondent’s consent to the test does not imply the knowledge of her HIV status.

However, respondents might have already taken an HIV test elsewhere through the available VCT services. Therefore, we had to consider the possible issue of reverse causality, with people making decisions on their partnership or parenthood based on the knowledge of their own HIV status. However, considering that at each study round, starting with the third one, respondents were asked whether they had ever thought about taking an HIV test, we managed to distinguish between those respondents who reported having already taken an HIV test (people who, most likely, knew their current HIV status), and those who declared that they never thought about taking the test or that they thought about taking it, but had not done it yet (S1 Text). We decided to exclude from the sample those individuals who were tested for HIV infection, reducing our sample to 7,905 respondents, of which 3,927 women and 3,978 men (S1 Table).

### Construction of event sequences

In order to construct the life course trajectories leading to parenthood, we considered the reported age (in years) when sexual debut (S), union–marriage, cohabitation or long-term relationship (more than twelve months) (U)–and parenthood (C) were firstly experienced. Information is available only about the year of age at the events. Therefore, when two or more events were experienced at the same year of age, we defined a combined event. For example, (SU) indicates that sexual debut and union occurred in the same year of age.

Each life course trajectory was described by the considered events in the order they were experienced for the first time. For example, the life course of a woman who had sexual debut at 17 years of age, married at 18 years, and had her first child at 20 years is represented by the ordered combination of the experienced events, the so-called *event sequence* (S)(U)(C). This sequence can be detailed by registering also the age at the events, obtaining the *time-and-event sequence* (S)(U)(C)(17,18,20). Whilst the number of event sequences is clearly limited, the number of time-and-event sequences is generally high, since each individual experiences the events at different ages. To simplify the latter sequences, one could refer to *event sequence analysis* [49,50], which defines suitable criteria to measure the *dissimilarity* between two time-and-event sequences. Cluster analysis can be applied to such dissimilarities, to obtain groups of individuals who are similar with respect both to the ordering and to the timing of the events. Unfortunately, in our case such approach led to unsatisfactory results (reported for completeness in S2 Text). First, the obtained clusters generally included individuals who experienced the same event sequence, without properly distinguishing between different time-and-event sequences. Second, the less frequent event sequences were not identified (even imposing a higher number of clusters), and they were rather assigned to clusters dominated by the most frequent sequences.

We therefore decided to focus only on the individuals’ event sequences (from now on, simply referred to as *sequences*), taking explicitly into account *all* the observed ordered combinations of events. The timing of events, which is clearly a relevant aspect of the life courses, was included in our analysis using the ages at the three events as independent variables in the statistical models for the association between HIV infection and sequences (illustrated in the next section).

Even when focusing only on the observed combinations, there is a relevant aspect to consider when dealing with time at event data. Indeed, some individuals presented *complete sequences* (i.e., they experienced all the considered events by the last interview). Instead, other individuals experienced only some events (at least by the last interview), and their life courses are consequently censored, since the inexperienced events might still occur later. Even so, the probability of experiencing such events clearly depends on the individual’s age at the time of the last interview. For example, consider two women, aged respectively 19 and 30 years at the moment of the last interview, who are characterised by the same sequence, (S)(U), and who experienced the two events at the same ages. For the first woman a problem of censoring arises, because she might become a mother after the last interview. Instead, the second woman had no children in the relatively large interval between her first union and the last observation period. Hence, her sequence can be reasonably considered as complete: the probability that she will give birth after the interview can be expected to be low.

To accommodate the incomplete sequences (thus limiting the problem of censoring) accounting for the individuals’ ages, we defined a new set of sequences built as follows. Consider the incomplete sequence (S)(U), which can clearly only evolve into (S)(U)(C). For each individual presenting the complete sequence (S)(U)(C), we derive δ_UC_, the observed waiting time from (U) to (C), and fit a generalised linear model (with gamma distribution and an inverse gamma link [51]) relating δ_UC_ to the age at (U). We then consider the residuals, ${\hat{\varepsilon }}_{UC,i}={(\delta }_{UC,i}-{\hat{\delta }}_{UC,i})$, and define the threshold ${\bar{\varepsilon }}_{UC}$ as the 90^th^ percentile of their distribution. Clearly, cases with a residual ${\hat{\varepsilon }}_{UC,i}>{\bar{\varepsilon }}_{UC}>0$ are characterised by a time to the last event, here (C), much higher than the predicted one, ${\hat{\delta }}_{UC,i}$, and thus experienced a *delayed* last event. Based on this reasoning, we further considered the time between the age at (U) and the age at the last interview (*T*_*F*_), $\delta_{UT_{F},i}$, and derived the predicted waiting time, ${\hat{\delta }}_{UT_{F},i}$, and the residual ${\hat{\varepsilon }}_{UT_{F},i}={(\delta }_{UT_{F},i}-{\hat{\delta }}_{UT_{F},i})$. Cases characterised by ${\hat{\varepsilon }}_{UT_{F},i}>{\bar{\varepsilon }}_{UC}>0$, were assigned the *delayed* sequence (S)(U) → (C). Such sequence indicates that, irrespective of the possible experience of event (C) after the last year of observation, these individuals delay childbearing compared to the other individuals in the sample with the same initial track. Conversely, we had to remove from the analysis those cases observed for a period shorter than ${\bar{\varepsilon }}_{UC}$, since the incompleteness of their sequence could not be accommodated.

The procedure just described was applied to all the incomplete sequences, using thresholds which, albeit different, all vary between 2 and 3 years. Note that the incomplete sequence (S) can evolve into different complete sequences, characterised by different waiting times between (S) and the subsequent event. Thus, to avoid an excess of intervention on data, we had to exclude cases presenting this specific sequence from the sample (S2 Table).

The final sample, selected and filtered as described in the previous sections, consisted of 5,307 individuals, of which 3,269 women and 2,011 men (S1 Table).

### Statistical analysis

We modelled the probability of being HIV-positive as a function of the obtained event sequences using logistic regression and adjusting for background socio-demographic effects. The individual HIV status was derived based on the application of the dipstick assay to the dried blood spots. The socio-demographic effects included in the model were birth cohort (1961–1970, 1971–1980, and 1981–1990), urban (small township) or rural site (estate, roadside settlement or subsistence farming area), current age of respondent at the last available round (included up to a quadratic term), and age at school leaving (a proxy for the socioeconomic information on the level of education). To account for the age at the focal events, the ages at first union and at the first child (set equal to zero for those individuals who did not experience such events) were also inserted into the model. We ran separate analyses for women and men, as we expected the sequences to have structural gender-based differences in terms of ordering and timing [9].

To explore the interaction between sequences and known transmission mechanisms for HIV, we also fitted two additional models including two intermediate HIV risk factors. Specifically, we considered the years since sexual debut (calculated as the difference between the current age of respondent and the age at first sexual intercourse) and the type of experienced non-regular sexual partnerships (none, only premarital–before the first union, only extramarital–after entering the first union, and both premarital and extramarital).

We also performed a robustness check to investigate the effect of the incomplete sequences on HIV infection, focusing only on individuals with complete trajectories (details and results are reported in S3 Text). This approach tends to exclude younger people, who are more likely to experience incomplete and therefore censored sequences. Even so, it provides useful additional insights: in the case when a delayed sequence is associated with HIV infection, it is possible to assess whether the association is driven by individuals who actually experienced the event with delay, or by those who never experienced it.

All data cleaning and analyses were performed using the software R. The algorithm to build the sequences was written using SAS.

## Results

### Sample description

As described before, our final sample consists of 3,269 women and 2,011 men, all sexually experienced, whose characteristics are reported in Tables 1 and 2, respectively. Men and women in the sample showed different socio-demographic patterns, mainly because of the older age of men (S3 Table). Included participants belonged mostly to the birth cohort 1971–1980 (43.1% of women vs. 52.8% of men, *P*<0.001), as reflected by the median age at last interview (28 years for women vs. 32 years for men, *P*<0.001). Moreover, women differed from men by living setting (19.1% of women living in an urban area vs. 23% of men, *P*<0.001), by age at school leaving (16 years old for women vs. 18 years old for men, *P*<0.001), and by HIV prevalence (28% of women vs. 24.1% of men, *P*<0.001). Prior research relates the latter difference to higher AIDS-related mortality among men [52,53], to higher women’s susceptibility to HIV infection [54], and to the possibility that women acquire HIV infection from committed partners who often also engage in extramarital sexual relationships [55]. Gender-based differences were also observed in the ages at events, with women showing a higher probability of experiencing events earlier than men, and in the exposure to non-regular sexual partnerships. In particular, the majority of women (63.8%) did not report any non-regular sexual relationship, whereas the majority of men (55.5%) reported at least one premarital sexual partner (S3 Table). Moreover, the proportion of women reporting both premarital and extramarital sexual partners was much lower than for men (2.5% for women vs. 22.4% for men, *P*<0.001), which might also be a consequence of social desirability bias [45–48].

**Table 1:** Characteristics of women included in the sample. Descriptive statistics on socio-demographic and sexual behaviour characteristics for women, stratified by sequence, and overall, Manicaland (Zimbabwe), 2000–2011. Sequences are sorted in descending order by HIV prevalence.

Sequence	N	HIV	Urban residence	Age at interview	Age at school leaving	Sexual debut		Union		Parenthood		Non-regular sexual partnerships			
						Age	S<15 yrs.	Ever in union	U<18 yrs.	Ever got child	C<18 yrs.	None	Premarital	Extramarital	Both
**(S)(U)->(C)**	56	54%	39%	32	16	17	11%	100%	21%	70%	0%	35%	48%	7%	9%
**(SC)->(U)**	15	53%	20%	30	17	20	0%	27%	0%	100%	27%	13%	60%	20%	7%
**(U)(S)->(C)**	6	50%	0%	34	18	19	0%	100%	67%	100%	0%	60%	0%	40%	0%
**(S)(C)(U)**	51	47%	25%	27	17	18	10%	100%	2%	100%	22%	20%	63%	6%	12%
**(SC)(U)**	38	37%	18%	29	18	19	0%	100%	3%	100%	24%	39%	37%	8%	16%
**(S)(C)->(U)**	44	36%	23%	32	17	18	11%	32%	0%	100%	14%	9%	72%	12%	7%
**(S)(UC)**	170	36%	26%	28	17	18	8%	100%	21%	100%	21%	49%	34%	13%	4%
**(SU)->(C)**	296	36%	19%	30	16	18	7%	100%	39%	75%	0%	61%	28%	8%	3%
**(S)(U)(C)**	309	33%	21%	28	17	17	11%	100%	26%	100%	12%	44%	39%	13%	5%
**(U)(SC)**	24	29%	17%	22	17	20	0%	100%	38%	100%	25%	91%	0%	9%	0%
**(SUC)**	519	25%	17%	28	17	19	0%	100%	25%	100%	25%	70%	18%	12%	1%
**(SU)(C)**	1701	24%	18%	27	16	18	4%	100%	40%	100%	15%	71%	18%	9%	2%
**(U)(S)(C)**	40	20%	12%	28	16	18	0%	100%	68%	100%	5%	92%	0%	8%	0%
**Total**	3269	28%	19%	28	16	18	5%	99%	34%	97%	16%	64%	24%	10%	2%

**Table 2:** Characteristics of men included in the sample. Descriptive statistics on socio-demographic and sexual behaviour characteristics for men, stratified by sequence, and overall, Manicaland (Zimbabwe), 2000–2011. Sequences are sorted in descending order by HIV prevalence.

Sequence	N	HIV	Urban residence	Age at interview	Age at school leaving	Sexual debut		Union		Parenthood		Non-regular sexual partnerships			
						Age	S<15 yrs.	Ever in union	U<18 yrs.	Ever got child	C<18 yrs.	None	Premarital	Extramarital	Both
**(S)(C)->(U)**	32	50%	28%	33	19	18	3%	53%	0%	100%	3%	0%	61%	6%	32%
**(U)(SC)**	2	50%	0%	38	20	29	0%	100%	0%	100%	0%	50%	0%	50%	0%
**(S)(U)->(C)**	188	43%	24%	36	18	18	11%	100%	4%	87%	0%	3%	59%	5%	32%
**(S)(C)(U)**	139	35%	28%	32	18	19	2%	100%	0%	100%	1%	4%	64%	2%	30%
**(S)(U)(C)**	859	23%	23%	31	18	19	3%	100%	2%	100%	0%	6%	64%	3%	26%
**(S)(UC)**	268	21%	23%	31	18	19	2%	100%	1%	100%	1%	6%	63%	3%	28%
**(SC)->(U)**	5	20%	20%	40	18	20	0%	100%	0%	100%	20%	20%	60%	0%	20%
**(SU)->(C)**	84	19%	18%	35	18	20	0%	100%	12%	87%	0%	35%	45%	7%	13%
**(SU)(C)**	333	17%	24%	29	18	22	0%	100%	8%	100%	1%	49%	33%	12%	6%
**(SUC)**	65	14%	18%	29	17	23	0%	100%	2%	100%	2%	58%	23%	12%	6%
**(SC)(U)**	16	13%	13%	31	18	21	0%	100%	0%	100%	6%	19%	50%	19%	13%
**(U)(S)(C)**	17	12%	12%	29	17	22	0%	100%	6%	100%	0%	59%	0%	41%	0%
**(U)(S)->(C)**	3	0%	33%	40	16	21	0%	100%	33%	100%	0%	100%	0%	0%	0%
**Total**	2011	24%	23%	32	18	19	3%	99%	4%	98%	1%	16%	56%	6%	22%

### Description of event sequences

Our results show that women and men experience similar sequences, characterised nonetheless by different frequencies and by different levels of HIV prevalence, which range from 20% to 54% for women (Table 1), and from 0 to 50% for men (Table 2). We decided to avoid the aggregation of low-frequency sequences with those with higher frequency, since this would lead to extremely heterogeneous groups of individuals in terms of both HIV prevalence and other important risk factors.

The predominant sequence for women is (SU)(C) (Table 1), characterised by a very short interval between the events, the lowest HIV prevalence (24%; average prevalence 28%), low levels of early sexual debut, moderate levels of early union and childbearing, and a low percentage of non-regular sexual partnerships (29%). This sequence can be considered as the normative transition to parenthood for women in Zimbabwe, as the average ages at events are consistent with those reported for the entire country [56]. On the other hand, we notice that this sequence, as well as the other sequences presenting a short span between sexual debut and union and very low proportions of non-regular sexual partnerships, report a relatively low prevalence, although actually not small in magnitude. This fact could be related to the sexual behaviour of their male partners [55], who, particularly when older than their spouses, might have been exposed for a longer time to HIV risk and have accumulated large numbers of non-regular sexual partners [12,57].

**Fig 1:**
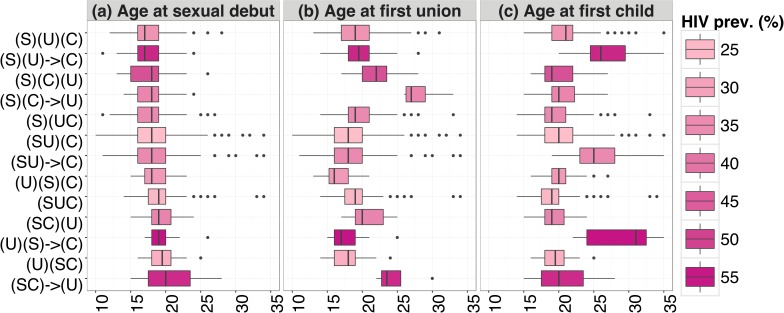
Age at events by sequence and HIV prevalence for women. Boxplot of the age at sexual debut, age at first union, and age at first child, by sequence, sorted by median age at sexual debut (in ascending order) and coloured according to the HIV prevalence, Manicaland (Zimbabwe), 2000–2011.

Conversely, sequences with higher HIV prevalence (above 35%) are generally characterised by a gap between sexual debut and first union, delayed (or inexperienced) union or motherhood, but also premarital pregnancy (Fig 1). More than one half of the women presenting these sequences show patterns of non-regular sexual intercourse (Table 1). In particular, women with both a gap between sexual debut and first union and premarital motherhood report a high average number of premarital sexual partners, around 2.4 (S4 Table).

**Fig 2:**
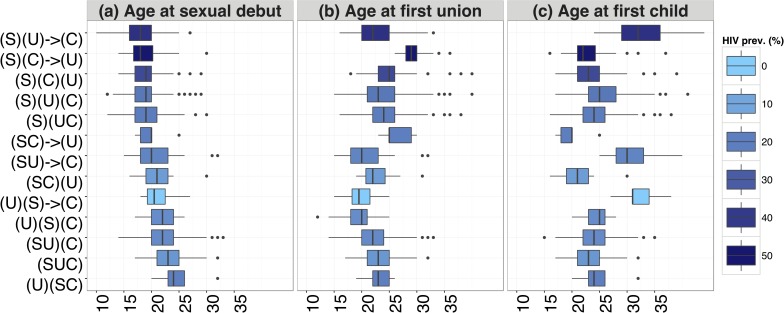
Age at events by sequence and HIV prevalence for men. Boxplot of the age at sexual debut, age at first union, and age at first child, by sequence, sorted by median age at sexual debut (in ascending order) and coloured according to the HIV prevalence, Manicaland (Zimbabwe), 2000–2011.

For men, the two most frequent sequences are (S)(U)(C) and (SU)(C), with HIV prevalence levels close to the average or lower (Table 2). Both the sequences are experienced by men younger than the average, but who differ in terms non-regular sexual partnerships. Indeed, a high percentage of men with the first sequence had premarital sexual partners, while almost half of the men with the second sequence never engaged in non-regular sex. On the other hand, in a similar way to what was observed for women, the sequences presenting the highest HIV prevalence (above 30%) are those characterised by delayed (or inexperienced) union or fatherhood, but also premarital parenthood (Fig 2). Moreover, most of men characterised by these high HIV-prevalent sequences experienced non-regular sex, with a high average number of premarital sexual partners (S4 Table), high rates of both premarital and extramarital sex [33], and long intervals between sexual debut and union (Fig 2).

### Relationship between event sequences and HIV infection

Table 3 reports the adjusted odds ratios from the logistic regression relating HIV infection to the explanatory variables for women. The most frequent sequence, (SU)(C), was used as a reference category. According to Model 1, four sequences show a significantly positive odds ratio, namely, (S)(U)(C), (S)(UC), (S)(C)(U), and (SC)(U), with the last two sequences presenting premarital childbearing. These sequences are characterised by an early sexual debut (except for (SC)(U), where sexual debut occurs later), and by a median gap between sexual debut and first union of one year, which increases to four years for (S)(C)(U) (Fig 1).

**Table 3:** Adjusted prevalence odds ratios for women. Adjusted odds ratios and 95% confidence intervals for the probability of being HIV-infected, based on logistic regression models, among women, Manicaland (Zimbabwe), 2000–2011. Sample size is varying because of missing values.

Variable	Category	Model 1	Model 2	Model 3
Sequence				
	(SU)(C))	1.00	1.00	1.00
	(SUC)	1.07 (0.82,1.39)	1.07 (0.82,1.4)	0.9984 (0.7612,1.3096)
	(SU) → (C)	0.9976 (0.6025,1.6517)	0.99 (0.59,1.64)	0.99 (0.59,1.66)
	(S)(U)(C)	1.42 (1.07,1.88)*	1.33 (0.94,1.88)	1.18 (0.83,1.68)
	(S)(U) → (C)	1.79 (0.83,3.84)	1.65 (0.74,3.69)	1.82 (0.78,4.22)
	(S)(UC)	1.77 (1.22,2.56)*	1.66 (1.1,2.52)*	1.51 (0.99,2.29)
	(S)(C)(U)	2.68 (1.39,5.13)*	2.33 (1.08,5.06)*	1.83 (0.82,4.04)
	(S)(C) → (U)	1.35 (0.35,5.3)	0.95 (0.17,5.43)	0.76 (0.13,4.43)
	(SC)(U)	2.12 (1.02,4.39)*	2 (0.94,4.24)	1.56 (0.72,3.35)
	(SC) → (U)	5.83 (0.56,60.98)	4.74 (0.42,53.83)	3.39 (0.29,39.2)
	(U)(SC)	1.62 (0.62,4.21)	1.73 (0.65,4.56)	1.31 (0.43,3.94)
	(U)(S)(C)	0.7 (0.31,1.58)	0.74 (0.32,1.71)	0.81 (0.35,1.89)
	(U)(S) → (C)	1.97 (0.35,11.03)	2.06 (0.37,11.55)	1.31 (0.18,9.28)
Birth cohort				
	1961–1970	1.00	1.00	1.00
	1971–1980	0.98 (0.71,1.35)	0.97 (0.7,1.35)	0.99 (0.71,1.38)
	1981–1990	0.69 (0.44,1.08)	0.69 (0.44,1.08)	0.73 (0.46,1.17)
Setting of residence				
	Rural	1.00	1.00	1.00
	Urban	1.73 (1.41,2.12)*	1.72 (1.4,2.12)*	1.63 (1.32,2.01)*
Age at interview		1.71 (1.51,1.95)*	1.65 (1.4,1.96)*	1.72 (1.45,2.05)*
Age at interview (squared)		0.992 (0.9901,0.994)*	0.992 (0.99,0.994)*	0.9922 (0.9902,0.9942)*
Age at school leaving		0.98 (0.95,1.02)	0.98 (0.95,1.02)	0.98 (0.95,1.02)
Age at first union		0.95 (0.89,1.02)	0.98 (0.87,1.1)	0.96 (0.85,1.09)
Age at first child		1.01 (0.95,1.08)	1.01 (0.95,1.08)	0.9956 (0.9321,1.0635)
Years since sexual debut			1.04 (0.93,1.16)	0.9919 (0.8847,1.1121)
Non-regular sexual relation				
	None			1.00
	Premarital			1.85 (1.5,2.28)*
	Extramarital			2.25 (1.72,2.96)*
	Both			2.54 (1.5,4.31)*
*N*		3036	3036	2989
BIC		3439	3446	3363

* *P*-value 0.05.

As for the socio-demographic factors, living in an urban area is associated with greater HIV risk, while birth cohort, age at school leaving, and age at first union or first child turned out to be not significant. A significant negative curvilinear effect of the age at interview shows that HIV risk peaks at around 35 years of age and drops afterwards. Considering that the predicted HIV-adjusted life expectancy in Zimbabwe was between 36 and 38 years for both women and men [52], the observed result can be regarded as the consequence of HIV/AIDS-related mortality.

The results obtained for men are displayed in Table 4. The sequence (SU)(C) was used as reference also in this case, because of its low HIV prevalence and high frequency. The sequences positively associated with HIV infection are (S)(U) → (C) and (S)(C)(U), characterised respectively by delayed fatherhood (as confirmed by the robustness check performed on the complete sequences, reported in S3 Text) and premarital fatherhood. Moreover, both of them present an early sexual debut (Fig 2), and a gap between sexual debut and first union. Protective factors from HIV infection are being of a younger birth cohort, living in a rural area, and having a longer education. In a similar way to what was observed for women, we did not find any significant evidence of association between HIV infection and the age at first union or at the first child. As for the age at interview, its curvilinear effect indicates that HIV risk increases up to 32 years of age and then drops. Again, such decrease can be a consequence of HIV/AIDS-related mortality [52].

**Table 4:** Adjusted prevalence odds ratios for men. Adjusted odds ratios and 95% confidence intervals for the probability of being HIV-infected, based on logistic regression models, among men, Manicaland (Zimbabwe), 2000–2011. Sample size is varying because of missing values.

Variable	Category	Model 1	Model 2	Model 3
Sequence				
	(SU)(C)	1.00	1.00	1.00
	(SUC)	0.71 (0.32,1.59)	0.73 (0.33,1.63)	0.75 (0.33,1.67)
	(SU) → (C)	0.9 (0.39,2.05)	0.86 (0.37,1.98)	0.86 (0.37,2)
	(S)(U)(C)	1.29 (0.91,1.83)	0.92 (0.62,1.38)	0.75 (0.48,1.16)
	(S)(U) → (C)	2.42 (1.28,4.6)*	1.73 (0.89,3.39)	1.52 (0.76,3.03)
	(S)(UC)	1.18 (0.75,1.84)	0.84 (0.52,1.38)	0.65 (0.38,1.1)
	(S)(C)(U)	1.98 (1.16,3.38)*	1.32 (0.73,2.38)	1.04 (0.56,1.92)
	(S)(C) → (U)	2.54 (0.8,8.02)	1.36 (0.41,4.54)	0.98 (0.29,3.37)
	(SC)(U)	0.65 (0.14,3.11)	0.56 (0.12,2.71)	0.47 (0.1,2.3)
	(SC) → (U)	0.69 (0.07,7.17)	0.48 (0.05,5.03)	0.39 (0.04,4.3)
	(U)(SC)	3.72 (0.22,62.41)	5.44 (0.3,97.32)	5.49 (0.29,104.92)
	(U)(S)(C)	0.73 (0.15,3.46)	0.79 (0.16,3.79)	0.86 (0.18,4.19)
	(U)(S) → (C)	-	-	-
Birth cohort				
	1961–1970	1.00	1.00	1.00
	1971–1980	0.47 (0.33,0.67)*	0.49 (0.34,0.69)*	0.5 (0.35,0.72)*
	1981–1990	0.46 (0.25,0.83)*	0.49 (0.27,0.9)*	0.51 (0.28,0.95)*
Setting of residence				
	Rural	1.00	1.00	1.00
	Urban	1.56 (1.22,2.01)*	1.57 (1.22,2.03)*	1.51 (1.17,1.95)*
Age at interview		1.67 (1.35,2.06)*	1.55 (1.25,1.93)*	1.54 (1.24,1.91)*
Age at interview (squared)		0.9931 (0.9901,0.996)*	0.993 (0.9901,0.996)*	0.9933 (0.9904,0.9963)*
Age at school leaving		0.93 (0.89,0.96)*	0.93 (0.9,0.97)*	0.93 (0.89,0.97)*
Age at first union		1.03 (0.97,1.09)	1.07 (1,1.14)*	1.08 (1.01,1.16)*
Age at first child		0.99 (0.93,1.05)	0.98 (0.92,1.04)	0.98 (0.92,1.04)
Years since sexual debut			1.08 (1.03,1.13)*	1.07 (1.02,1.12)*
Non-regular sexual relation				
	None			1.00
	Premarital			1.56 (1.01,2.41)*
	Extramarital			1.52 (0.84,2.74)
	Both			2.37 (1.47,3.8)*
*N*		1937	1937	1918
BIC		2080	2077	2066

* *P*-value 0.05.

### Interaction between event sequences and HIV proximate determinants

We found that the length of sexual exposure and the type of non-regular sexual partnerships allow to understand the association between HIV risk and the sequences. Indeed, when these two additional variables are included in the model, most of the significant associations between sequences and HIV risk observed in Model 1 are no longer significant.

For women, there is no significant association between the length of the sexual exposure and HIV infection (Model 2, Table 3). Nonetheless, its inclusion in the model leads to a loss of the statistical significance for the sequences (S)(U)(C) and (SC)(U).

Conversely, a longer sexual exposure for men is associated with a higher HIV risk (Model 2, Table 4). When the variable is included, the association between HIV infection and both sequences (S)(C)(U) and (S)(U) → (C) is no longer significant. This may indicate that a long sexual exposure, likely initiated at an early age (Fig 2), moderates the association between the sequences and the HIV infection.

Nonetheless, the most relevant proximate determinant turns out to be the type of non-regular sexual relationships: its inclusion in Model 3 causes the loss of statistical significance for all sequences. For women, the experience of non-regular sex is an important transmission mechanism, with its odds ratios increasing in magnitude from premarital to extramarital sex, up to the combination of both types (Table 3). Along with the loss of significance, we observe a shrinking in the values of the odds ratios. This suggests that the experience of non-regular sexual relationships may explain part of the association between the sequences and HIV infection.

For men, we find a different pattern of association between non-regular sex and HIV infection. Indeed, the large majority of men experienced premarital sexual relationships, either alone or followed by extramarital relationships (Table 2). The high rates of non-regular sex, in combination with a longer exposure to sexual activity, produce the shrinking in the magnitude of the odds ratios, and this might explain the significance of the sequences in Model 1 (Table 4).

### Sequences and HIV infection across birth cohorts: a sign of behaviour change?

**Fig 3:**
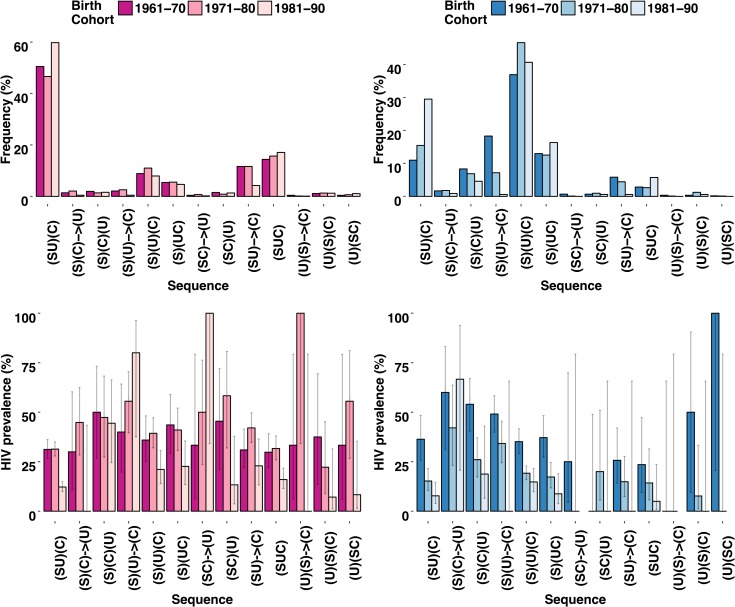
Relationship between sequences and HIV across birth cohorts. Change in the distribution across birth cohorts of the sequences and of their HIV prevalence, Manicaland (Zimbabwe), 2000–2011. Top row: bar plot of the distribution of sequences across birth cohorts, for women (left panel) and for men (right panel). Bottom row: bar plot (with 95% confidence interval) of the HIV prevalence associated with each sequence across birth cohorts, for women (left panel) and for men (right panel).

In our models, birth cohort was inserted as a control variable. Nonetheless, the relevance of the different sequences might vary for different birth cohorts, and this could explain the secular decline in HIV in Zimbabwe from 24% to 14% over the study period [37]. Therefore, we investigated the possible change in the sequences’ distribution across the different birth cohorts, and studied the relationship between such differences and HIV infection (Fig 3). We noticed that, for both women and men, the frequency of normative and “safer” sequences, e.g., (SU)(C) and (SUC), increased over birth cohorts, while the reverse holds for less normative sequences, such as (S)(U) → (C), (S)(C) → (U), or (SU) → (C) (Fig 3, top row). Interestingly, we also found that the HIV prevalence characterising normative and safer sequences showed a decreasing trend over cohorts (Fig 3, bottom row). This might be due to a change in the sexual behaviour across birth cohorts, which favoured more normative and less risky transitions to parenthood (e.g., (SU)(C) and (SUC)), with a consequent decrease in the HIV prevalence. On the other hand, we observed that for some less normative and riskier sequences, e.g., (S)(U) → (C) and (SC) → (U) for women, or (S)(C) → (U) for men, the associated HIV prevalence rose, despite its decrease in frequency across cohorts. Even though this change was not statistically significant (as indicated by the confidence intervals in the bar plots reported in Fig 3, bottom row), still it might provide some evidence of the risk associated in more recent times with non-normative transitions to parenthood and to adulthood, in particular those characterised by delayed or inexperienced union or parenthood, or premarital parenthood.

## Discussion

This was a study on the role of life course events during the transition to parenthood in determining the risk of HIV acquisition, for both women and men. In particular, our goal was to discriminate between those sequences that likely protected people from experiencing HIV infection, and those that, conversely, led to a higher chance of adopting risky sexual behaviours and, consequently, of acquiring HIV infection. Overall, we found evidence for associations between HIV infection and sequences, mostly due to their interplay with known mechanisms for HIV transmission, namely, a longer sexual exposure (possibly combined with early sexual debut), and engagement in non-regular sexual relationships. We also found that the extent of the association between demographic events–such as entering the first union or having the first child–and HIV infection depends primarily on their order, and only secondarily on the age of occurrence.

For women, sequences significantly associated with higher HIV prevalence are those diverging from the normative sequence (SU)(C), in particular, those with a gap between sexual debut and first union, namely, (S)(U)(C) and (S)(UC), and those with premarital motherhood, i.e., (S)(C)(U) and (SC)(U). Since the precise timing of HIV seroconversion (before or after pregnancy) is unknown, we cannot infer any causal link. Even so, some possible explanations for these associations can be suggested. First, longer periods of sexual activity before union, possibly combined with an early sexual debut, may lead to higher rates of premarital sex (S4 Table), and thus increase the risk of getting HIV-infected [16,20]. Second, since a high frequency of premarital sex is often related to a larger number of sexual partners [58], there is a higher chance of experiencing premarital motherhood [59]. This is in line with current trends that see more SSA women entering stable unions at later ages [60], and thus carrying prolonged risk of premarital birth [61]. Moreover, although most studies have found that worries of being HIV-positive reduce the desire to have children [62], nonetheless, in some cases, perceived positive HIV status is associated with stronger fertility intentions, as the idea of having children is an indication of “normal” life [17,63], also at the cost of experiencing it out of a stable union. However, this can be a further problem for the single mother, considering that, in many SSA countries, premarital pregnancy, although it might be seen as a sign of proven fertility, is still associated with issues such as socioeconomic vulnerability, stigmatisation, and health risk, both for the mother and for the child [61,64–68].

For men, we found that high HIV risk is associated with both delayed fatherhood, (S)(U) → (C), and premarital fatherhood, (S)(C)(U). Considering that these individuals report, on average, large numbers of premarital (S4 Table) or extramarital sexual partners, a “sexually adventurous” personality type, described as someone who is always drawn to the acquisition of new partners [20], could be inferred for them. As regards delayed fatherhood, this can be either a premise for, or the consequence of, HIV infection. On the one hand, it can be due to sub-fertility within the couple prior to HIV infection [69]. In this case, considering the social stigma associated with childlessness, the desire of the man to have biological children in a pronatalist society might result in risky practices, such as engagement in frequent extramarital sex [70]. On the other hand, delayed fatherhood might be a consequence of undiagnosed HIV infection, and be due, for example, to biological causes, such as a depressed immune system, weight loss, stress, but also the co-infection with other sexually transmitted diseases (STDs) [71,72]. Also situational factors, such as migratory patterns associated with work, might have an impact, as they can temporarily interrupt marital relations [21], with the consequent possible increase in the probability of engaging in extramarital relationships.

The described classification of sequences into those considered as normative and safer and those considered as non-normative and riskier leads to questions about their distribution and their association with HIV infection, over time and, in particular, across birth cohorts. We found that, irrespective of gender, the younger cohorts are more prone to experience normative sequences, such as (SU)(C) and (SUC), and, at the same time, show a lower HIV prevalence. This is in line with the estimated reduction in the HIV prevalence from the 24% at the beginning of the study (1998–2000) to the 14% at the time of the last study round (2011) [37], and is consistent with a reduction in casual sex and of multiple sexual relationships, reported elsewhere [36,38]. Even so, although the non-normative sequences are less frequent within the younger cohorts, the associated HIV risk still remains high and thus of great concern.

Our study suffers from some limitations that are worth discussing. First, given the structure of the survey, HIV prevalence (rather than incidence) was observed throughout the study and the precise timing at HIV seroconversion was unknown for most of the selected individuals, with a considerable proportion of subjects entering the study being already infected. Although the lack of such information makes the investigation of the causal relationships between HIV risk and life course trajectories impossible, individuals in our sample reported not having ever taken an HIV test, and the result of the HIV test conducted in the Manicaland HIV Study was not fed back to participants. It is therefore reasonable to expect that respondents did not make decisions about their life course events based on the knowledge of their HIV status (S1 Text). However, a person’ suspicion about his own HIV status or about the possible risk associated to his behaviour might nevertheless influence his decisions on life course events. There is some literature on local initiatives for HIV prevention taken by people to face the AIDS epidemic, such as discussing with other people the dangers related to risky sexual behaviours and HIV infection, up to the separation from partners who keep having unsafe sexual behaviours. The Manicaland HIV Prevention Study itself also produces results sheets for each study site, which are either provided to respondents in the next survey round, or used to disseminate the study results (including gaps in knowledge about HIV) at public meetings held in several locations in each study site, in order to increase HIV awareness. These results sheets are also given to local National AIDS Council and Ministry of Health officials for use in their HIV prevention programmes. As a matter of fact, evidence suggests that HIV awareness, opinions about what is socially considered as a “safe” or as a “risky” behaviour, as well as the fear of illness and death due to AIDS among relatives and social acquaintances, may drive people’s decisions on important life course events, even when the HIV status is unknown [17,63,73–75]. Further research could employ data on individuals’ perceptions and HIV awareness to better investigate the relationship between life course events and HIV infection. A second limitation of our study concerns the fact that only sexual intercourse was considered as an HIV transmission mode [13,76], thus disregarding other non-sexual routes, such as injecting drug use, blood contamination (e.g., through blood transfusions), unsafe medical injections, and mother-to-child transmission (MTCT), all of which are not influenced by the studied demographic events. Finally, other important socio-demographic and sexual behavioural factors, such as the socioeconomic status and use of condoms, might, in principle, have an effect on the relationship between the sequences and HIV infection. Even though several measures of socio-economic status and condom use are reported in the data, it was not possible to properly account for such factors in our particular analysis, because we did not have any information for the focal period of interest, namely, when the transition to parenthood was under way.

## Conclusions

The use of the event sequence framework allowed us to find that the timing of childbearing with respect to the timing of first union has a strong impact on HIV infection. Specifically, irrespective of gender, both the fact of experiencing parenthood outside the union or late within the union are associated with greater HIV risk. We also found that normative and safer sequences have become more common among younger birth cohorts, and are characterised by a lower HIV prevalence. This might be related to the change in sexual behaviour that has led to the observed reduction in HIV prevalence in the country in more recent years. On the other hand, riskier and non-normative sequences, notwithstanding the lower frequency within the younger birth cohorts, still present high HIV prevalence rates and remain of high concern.

Our findings may be particularly important to target existing and new HIV prevention methods. On the one hand, it would be important to promote condom use and pre-exposure prophylaxis to young unmarried women who have already experienced pregnancy and who may continue to practice unprotected sex in the hope of finding a marriage partner. For men, instead, early voluntary medical male circumcision and strengthening and intensifying the promotion of STD management programmes remain among the main strategies for reducing HIV and STD risk at each stage of the various possible trajectories, particularly for those men with non-normative sequences and a large number of non-regular sexual partners. Moreover, for men delaying fatherhood because of sub-fertility issues, improving health services and counselling might reduce the HIV risk associated with unhealthy sexual practices one can resolve to in order to finally have children.

## Supporting Information

S1 Data Final data for the analysis. Data set for women and men, without data inconsistencies and with individuals who have never taken an HIV test before.
(XLSX)

S1 Table Final sample. Determination of the final sample for the analysis.
(DOCX)

S2 Table Original and final sequences by gender. Comparison between original and final sequences, by gender.
(DOCX)

S3 Table Comparison between individuals included and excluded from the analysis, by gender. Descriptive statistics for individuals included in the analysis sample and excluded because their sequences could not be accommodated.
(DOCX)

S4 Table Premarital sex partners per sequence, by gender. Median number of premarital sexual partners per sequence, by gender.
(DOCX)

S1 Text Sequences and HIV infection by HIV testing history. Document with a descriptive analysis of the relationship between the event sequences and the HIV testing history of participants.
(DOCX)

S2 Text Event Sequence Analysis and Cluster Analysis. Document with the event sequence analysis applied to the time-and-event sequences, mentioned in the “Construction of event sequences” subsection.
(DOCX)

S3 Text Robustness check. Document with the analysis of the complete sequences only, performed as robustness check.
(DOCX)
