# Supplementary material for: Transition to Parenthood and HIV Infection in Rural Zimbabwe
Source: PLoS One. 2016 Sep 29;11(9):e0163730. doi: 10.1371/journal.pone.0163730 (PMC5042509; doi:10.1371/journal.pone.0163730)
Supplement: S1 Table — Determination of the final sample for the analysis. (DOCX) [file pone.0163730.s002.docx]

**Table A. Final sample.** Determination of the final sample for the analysis, Manicaland (Zimbabwe), 2000-2011.

| **Sample** | **Issue** | **Total** | **Women** | **Men** |
| --- | --- | --- | --- | --- |
| Eligible^*^ |  | 27,073 | 15,949 | 11,124 |
|  | *Inconsistencies in data^**^* | *15,436* | *9,130* | *6,306* |
| Partial sample |  | 11,647 | 6,829 | 4,818 |
|  | *HIV test already taken^***^* | *3,742* | *2,902* | *840* |
| Partial sample |  | 7,905 | 3,927 | 3,978 |
|  | *Censored sequences not accommodated^****^* | *2,625* | *658* | *1,967* |
| Final sample |  | 5,280 | 3,269 | 2,011 |

^*^ Eligibility criteria are: (i) being sexually experienced at the time of the last interview; (ii) having provided information on HIV testing history; (iii) having provided a dried blood specimen for HIV testing and having a clear test result.

^**^ Inconsistencies concern different years of birth or ages at events per subject across rounds, a declared age at first child lower than the declared age at sexual debut, a declared age at first union lower than the declared age at sexual debut, together with a declared positive number of premarital sexual partners.

^***^These are subjects who reported to have taken already an HIV test and thus are likely aware of their HIV status. They have been excluded from the sample because of possible reverse causality issues, i.e., when the experienced sequence is influenced by the knowledge of the HIV status.

^****^These are subjects showing a censored sequence that cannot be ruled out as incomplete, but rather could still likely evolve into a complete/incomplete sequence. For this reason, they had to be excluded from the sample.
