# Supplementary material for: Transition to Parenthood and HIV Infection in Rural Zimbabwe
Source: PLoS One. 2016 Sep 29;11(9):e0163730. doi: 10.1371/journal.pone.0163730 (PMC5042509; doi:10.1371/journal.pone.0163730)
Supplement: S2 Table — Comparison between original and final sequences, by gender. (DOCX) [file pone.0163730.s003.docx]

**Table B. Original and final sequences by gender.** Comparison between original and final sequences by gender, with the respective frequency and HIV prevalence.

| **Original**  **sequence** | **Final**  **sequence** | **Women** | | **Men** | |
| --- | --- | --- | --- | --- | --- |
|  |  | **N** | **HIV (%)** | **N** | **HIV (%)** |
| $(S)$ | - | 218 | 12.8% | 1641 | 4.6% |
|  |  |  |  |  |  |
| $(S)(C)$ | $\left( S \right)\left( C \right)\to(U)$ | 30 | 40% | 15 | 53.3% |
| $(S)(C)$ | - | 61 | 19.7% | 37 | 16.2% |
| $(S)(C)(U)$ | $(S)(C)\to(U)$ | 14 | 28.6% | 17 | 47.1% |
| $(S)(C)(U)$ | $\left( S \right)\left( C \right)(U)$ | 51 | 47.1% | 139 | 35.3% |
|  |  |  |  |  |  |
| $(S)(U)$ | $\left( S \right)\left( U \right)\to(C)$ | 17 | 58.8% | 24 | 45.8% |
| $(S)(U)$ | - | 53 | 18.9% | 194 | 12.4% |
| $(S)(U)(C)$ | $\left( S \right)\left( U \right)\to(C)$ | 39 | 51.3% | 164 | 42.1% |
| $(S)(U)(C)$ | $\left( S \right)\left( U \right)(C)$ | 309 | 33.3% | 859 | 22.6% |
|  |  |  |  |  |  |
| $(S)(UC)$ | $\left( S \right)\left( \mathrm{UC} \right)$ | 170 | 35.9% | 268 | 21.3% |
|  |  |  |  |  |  |
| $(SC)$ | $\left( \mathrm{SC} \right)\to(U)$ | 11 | 45.5% | 0 | - |
| $(SC)$ | - | 18 | 11.1% | 4 | 0% |
| $(\mathrm{SC})(U$) | $\left( \mathrm{SC} \right)\to(U)$ | 4 | 75% | 5 | 20% |
| $(SC)(U)$ | $\left( \mathrm{SC} \right)(U)$ | 38 | 36.8% | 16 | 12.5% |
|  |  |  |  |  |  |
| $(SU)$ | $\left( \mathrm{SU} \right)\to(C)$ | 74 | 45.9% | 11 | 18.2% |
| $(SU)$ | - | 299 | 11.4% | 88 | 11.4% |
| $(SU)(C)$ | $(SU)(C)$ | 1701 | 23.7% | 333 | 17.1% |
| $(SU)(C)$ | $\left( \mathrm{SU} \right)\to(C)$ | 222 | 32.4% | 73 | 19.2% |
|  |  |  |  |  |  |
| $(SUC)$ | $(SUC)$ | 519 | 25.4% | 65 | 13.8% |
|  |  |  |  |  |  |
| $(U)(S)$ | - | 9 | 22.2% | 3 | 0% |
| $(U)(S)(C)$ | $\left( U \right)\left( S \right)\to(C)$ | 6 | 50% | 3 | 0% |
| $(U)(S)(C)$ | $\left( U \right)\left( S \right)(C)$ | 40 | 20% | 17 | 11.8% |
|  |  |  |  |  |  |
| $(U)(SC)$ | $(U)(SC)$ | 24 | 29.2% | 2 | 50% |
|  |  | 3,927 |  | 3,978 |  |
