# Supplementary material for: Transition to Parenthood and HIV Infection in Rural Zimbabwe
Source: PLoS One. 2016 Sep 29;11(9):e0163730. doi: 10.1371/journal.pone.0163730 (PMC5042509; doi:10.1371/journal.pone.0163730)
Supplement: S3 Table — Descriptive statistics for individuals included in the analysis sample and excluded because their sequences could not be accommodated. (DOCX) [file pone.0163730.s004.docx]

**Table C. Comparison between individuals included and excluded from the analysis, by gender.** HIV prevalence, socio-demographic and sexual behaviour risk factors, and timing of life course events for men and women, either included in the analysis sample or excluded because their sequences could not be accommodated. Manicaland (Zimbabwe), 2000-2011.

| **Variable** | **Category** | **Men** | | | **Women** | | |
| --- | --- | --- | --- | --- | --- | --- | --- |
|  |  | **In sample** | **Out sample** | ***P*** | **In sample** | **Out sample** | ***P*** |
| N |  | 2,011 | 1,967 |  | 3,269 | 658 |  |
| HIV status |  |  |  | <0.001 |  |  | <0.001 |
|  | *HIV-negative* | 1,527 (75.9%) | 1,851 (94.1%) |  | 2,354 (72%) | 570 (86.6%) |  |
|  | *HIV-positive* | 484 (24.1%) | 116 (5.9%) |  | 915 (28%) | 88 (13.4%) |  |
| Birth cohort |  |  |  | <0.001 |  |  | <0.001 |
|  | *1961–1970* | 601 (29.9%) | 19 (1%) |  | 723 (22.1%) | 5 (0.8%) |  |
|  | *1971–1980* | 1,061 (52.8%) | 522 (26.5%) |  | 1,410 (43.1%) | 92 (14%) |  |
|  | *1980–1990* | 349 (17.4%) | 1,426 (72.5%) |  | 1,136 (34.8%) | 561 (85.2%) |  |
| Setting of residence |  |  |  | 0.033 |  |  | 0.69 |
|  | *Rural* | 1,548 (77%) | 1,570 (79.8%) |  | 2,643 (80.9%) | 527 (80.1%) |  |
|  | *Urban* | 463 (23%) | 397 (20.2%) |  | 626 (19.1%) | 131 (19.9%) |  |
| Type of non-regular sex |  |  |  | <0.001 |  |  | <0.001 |
|  | *None* | 323 (16.2%) | 85 (4.4%) |  | 2,052 (63.8%) | 265 (40.7%) |  |
|  | *Only premarital* | 1,106 (55.5%) | 1,615 (82.9%) |  | 767 (23.8%) | 262 (40.2%) |  |
|  | *Only extramarital* | 117 (5.9%) | 64 (3.3%) |  | 318 (9.9%) | 103 (15.8%) |  |
|  | *Both* | 446 (22.4%) | 185 (9.5%) |  | 79 (2.5%) | 21 (3.2%) |  |
| Age at interview^1^ |  | 32 [27-37] | 22 [20-25] | <0.001 | 28 [23-34] | 20 [19-23] | <0.001 |
| Age at school leaving^1^ |  | 18 [17-19] | 18 [17-19] | <0.001 | 16 [15-18] | 17 [15-18] | <0.001 |
| Age at sexual debut^1^ |  | 19 [17-22] | 18 [16-20] | <0.001 | 18 [17-20] | 18 [17-20] | <0.001 |
| Age at first union^1^ |  | 23 [21-25] | 23 [21-25] | <0.001 | 18 [17-20] | 19 [17-21] | <0.001 |
| Age at first child^1^ |  | 25 [23-28] | 22 [20-23] | <0.001 | 20 [18-22] | 20 [18-22] | <0.001 |

^1^ Median with 3^rd^ and 4^th^ quartiles.
