# Supplementary material for: Transition to Parenthood and HIV Infection in Rural Zimbabwe
Source: PLoS One. 2016 Sep 29;11(9):e0163730. doi: 10.1371/journal.pone.0163730 (PMC5042509; doi:10.1371/journal.pone.0163730)
Supplement: S4 Table — Median number of premarital sexual partners per sequence, by gender. (DOCX) [file pone.0163730.s005.docx]

**Table D. Premarital sex partners per sequence, by gender.** Median number of premarital sexual partners per sequence, by gender. Sequences are ordered by HIV prevalence per sequence for women.

| **Sequence** | **Women** | | | **Men** | | |
| --- | --- | --- | --- | --- | --- | --- |
|  | **N** | **HIV (%)** | **No. premarital**  **sex partners** | **N** | **HIV (%)** | **No. premarital**  **sex partners** |
| **(S)(U)->(C)** | 56 | 54% | 1 | 188 | 43% | 2 |
| **(SC)->(U)** | 15 | 53% | 1 | 5 | 20% | 4 |
| **(U)(S)->(C)** | 6 | 50% | 0 | 3 | 0% | 0 |
| **(S)(C)(U)** | 51 | 47% | 1 | 139 | 35% | 3 |
| **(SC)(U)** | 38 | 37% | 1 | 16 | 13% | 1 |
| **(S)(C)->(U)** | 44 | 36% | 1 | 32 | 50% | 4 |
| **(S)(UC)** | 170 | 36% | 0 | 268 | 21% | 2 |
| **(SU)->(C)** | 296 | 36% | 0 | 84 | 19% | 1 |
| **(S)(U)(C)** | 309 | 33% | 0 | 859 | 23% | 2 |
| **(U)(SC)** | 24 | 29% | 0 | 2 | 50% | 0 |
| **(SUC)** | 519 | 25% | 0 | 65 | 14% | 0 |
| **(SU)(C)** | 1,701 | 24% | 0 | 333 | 17% | 0 |
| **(U)(S)(C)** | 40 | 20% | 0 | 17 | 12% | 0 |
| **Total** | 3,269 | 28% | 0 | 2,011 | 24% | 2 |
