# Supplementary material for: Transition to Parenthood and HIV Infection in Rural Zimbabwe
Source: PLoS One. 2016 Sep 29;11(9):e0163730. doi: 10.1371/journal.pone.0163730 (PMC5042509; doi:10.1371/journal.pone.0163730)
Supplement: S1 Text — Document with a descriptive analysis of the relationship between the event sequences and the HIV testing history of participants. (DOCX) [file pone.0163730.s006.docx]

**S1 Text**

**Sequences and HIV infection by HIV testing history**

In order to explore the association between sequences and the possible knowledge about the HIV status, we made the plot reporting, for each sequence, the distribution of the answers to the question “Have you ever taught to take an HIV test?” (with possible answers: never thought, thought about taking it, but not done yet, took an HIV test), irrespective of the results of the HIV test performed with the dipstick assay during the study.

With the exception of the sequences where first union occurs before sexual debut, for all the other sequences a quite similar distribution of the three categories of access to voluntary HIV testing can be observed. The bar plots show that, for the majority of sequences, most of the individuals never thought about taking an HIV test or, if they thought about it, they had not taken it yet. More normative sequences, like those without a gap between sexual debut and first union, and no delayed union or parenthood, show higher rates of HIV test uptake. Despite this, and with the mentioned exceptions, no evidence of particular patterns was found across sequences.

Figure A Relationship between sequences and HIV by HIV testing history. Change in the distribution between those who never took an HIV test, those who thought about taking it, but have not done it yet, and those who already did it (the latter ones are excluded from the sample in the main analysis), of sequences and of their HIV prevalence, Manicaland (Zimbabwe), 2000-2011. Left panel: bar plot of the distribution of sequences by HIV testing history for women. Right panel: bar plot of the distribution of sequences by HIV testing history for men.
