# Supplementary material for: Transition to Parenthood and HIV Infection in Rural Zimbabwe
Source: PLoS One. 2016 Sep 29;11(9):e0163730. doi: 10.1371/journal.pone.0163730 (PMC5042509; doi:10.1371/journal.pone.0163730)
Supplement: S2 Text — Document with the event sequence analysis applied to the time-and-event sequences, mentioned in the “Construction of event sequences” subsection. (DOCX) [file pone.0163730.s007.docx]

**S2 Text**

**Event Sequence Analysis and Cluster Analysis**

**Methods**

Event sequence analysis techniques [1] were applied to the original event-and-time sequences (from now, just called sequences) to obtain clusters of sequences that were similar both in terms of ordering and timing of the events. The analysis was performed using the R package *TraMineR* [2].

In brief, the routine procedure consists in mapping each sequence to any other individual sequence using a series of transformations, such as the insertion/deletion of an event and the translation of an event by a unit of time. To each transformation, a cost chosen by the researcher is assigned. The effect of the insertion/deletion costs is to give more importance to the ordering of the events experienced by individuals; instead, the translation costs impact on the timing of the events, so that the higher the cost, the more influential a delay in the timing of a certain event. Using these costs, we can compute the dissimilarities between pairs of sequences employing, for instance, the *optimal matching edit* distance, defined as the minimum cost necessary to transform a sequence into another one [1].

These dissimilarities can be used to obtain clusters, possibly uncovering the hidden structures in the sequences. Here, we opted for a method that combined hierarchical clustering with partition-based clustering, namely, a *Partitioning Around Medoid* clustering approach, which is based on the *k*-group solution from a hierarchical clustering with Ward’s method [1]. The final solution, i.e., the optimal number of clusters, was selected using the *overall average silhouette width* [3], according to which, the higher this measure, the better pronounced the differences between clusters, and thus the better the solution.

**Results**

By applying event sequence analysis and cluster analysis to the original sequences, we obtained four clusters of sequences for women, and seven clusters for men.

Table A reports, among other information, the medoid of each cluster (i.e., the sequence having the smallest average distance from the other sequences in the cluster). Looking carefully at the clusters’ compositions, we observed that some clusters include sequences that are quite dissimilar from the corresponding medoid, and that some sequences with similar characteristics were assigned to different clusters. For instance, the combination of events $\text{(SC)}$, which is uncommon for both women and men, is included in two different clusters for women, namely, those with medoid $(S)\left( U)(C \right)$ (18,19,21 | $T_{F}$ = 35) and $(S)\left( \mathrm{UC} \right)$ (18,19 | $T_{F}$ = 26). A similar situation was observed for men, namely, the combination $\text{(SC)}$ was assigned to the clusters with medoid $\left( S)(C \right)(U)$ (18,23,25 | $T_{F}$ = 35) and $\left( S)(U \right)$ (18,23 | $T_{F}$ = 23).

Moreover, comparing Table A with Tables 1-2 in the main manuscript, we can notice that these cluster solutions hide some particular sequences characterised by a very high HIV prevalence (>40%), such as $\left( S \right)\left( U \right)\to(C)$, for both women and men.

In conclusion, since (i) clusters are dominated by the most frequent sequences in terms of ordering of the events, independently of the timing, (ii) the less frequent sequences are assimilated to other sequences that are quite different from them, and (iii) sequences with very high HIV prevalence are hidden among sequences with quite different HIV prevalence, we decided to follow a different approach. This new approach, which is the one implemented in the main manuscript, prioritise the ordering of the events over their timing, and thus considers all the possible observed sequences in terms of ordering, and their association with HIV infection.

**Table A. Clusters of sequences by gender.** Characteristics of the clusters of sequences, sorted by mean HIV prevalence in descending order, for women and men, respectively, with cluster size (N) and median age at last round.

| **Women** | | | |
| --- | --- | --- | --- |
| Medoid | N | HIV (%) | Age |
| $\left( S \right)(UC)$ (18,19 \| $T_{F}$ = 26) | 313 | 29.4 | 28 |
| $\left( S \right)(U)(C)$ (18,19,21 \| $T_{F}$ = 35) | 1394 | 26.4 | 25 |
| $(SUC)$ (19 \| $T_{F}$ = 20) | 969 | 22.5 | 28 |
| $\left( \mathrm{SU} \right)(C)$ (18,20 \| $T_{F}$ = 30) | 4147 | 21.9 | 27 |
| **Men** | | | |
| Medoid | N | HIV (%) | Age |
| $\left( S \right)(C)(U)$ (18,23,25 \| $T_{F}$ = 35) | 278 | 28.4 | 32 |
| $\left( S \right)(U)(C)$ (19,23,26 \| $T_{F}$ = 35) | 1343 | 25.8 | 32 |
| $\left( S \right)(UC)$ (19,24 \| $T_{F}$ = 35) | 340 | 20.6 | 31 |
| $\left( \mathrm{SU} \right)(C)$ (22,25 \| $T_{F}$ = 25) | 645 | 17.4 | 29 |
| $\left( S)(U \right)$ (18,23 \| $T_{F}$ = 23) | 313 | 14.7 | 26 |
| $(SUC)$ (23 \| $T_{F}$ = 35) | 93 | 10.8 | 29 |
| $(S)$ (18 \| $T_{F}$ = 18) | 1795 | 4.8 | 22 |

**References**

1. Studer M, Müller NS, Ritschard G, Gabadinho A. Classer, discriminer et visualiser des séquences d'événements. RNTI. EGC; 2010;E-19: 37–48.

2. Ritschard G, Burgin R, Studer M. Exploratory Mining of Life Event Histories. In: McArdle JJ, Ritschard G, editors. *Contemporary Issues in Exploratory Data Mining in the Behavioral Sciences*. New York (NY): Routledge; 2013. pp. 221–253.

3. Rousseeuw PJ. Silhouettes: a graphical aidto the interpretation and validation of cluster analysis. J Comput Appl Math. 1987;20: 53–65.
