# Supplementary material for: Transition to Parenthood and HIV Infection in Rural Zimbabwe
Source: PLoS One. 2016 Sep 29;11(9):e0163730. doi: 10.1371/journal.pone.0163730 (PMC5042509; doi:10.1371/journal.pone.0163730)
Supplement: S3 Text — Document with the analysis of the complete sequences only, performed as robustness check. (DOCX) [file pone.0163730.s008.docx]

**S3 Text**

**Robustness checks**

**Analysis of Original Complete Sequences**

In addition to the analysis presented in the main manuscript, we performed a robustness check to evaluate thoroughly our findings. As mentioned in the methodology section of the manuscript, alternative strategies can be followed to deal with incomplete sequences, which may be censored or not, so that possible associations between certain life course sequences and HIV prevalence could be more easily identified. In particular, in this section we focus on one alternative different approach.

Specifically, we limit the attention to individuals with complete sequences, who therefore experienced all the three considered events in whatever order and timing. This is a sort of complete-case analysis that excludes individuals reporting only one or two of the three considered events. In our case, this action leads to the removal from the sample of the youngest cohorts. Also, this procedure can lead to possible bias, since some of the removed *incomplete* sequences may actually be complete in the sense that some individuals may *decide* not to experience some events (e.g., entering a union or having children).

Tables A-B report the results of Model 1 and Model 3 limited to the complete sequences, for women and men, respectively. As expected, the analysed individuals (whose median ages at the last interview are equal to 28 and 32 years, for women and men, respectively) are older compared to the whole sample (with median ages equal to 26 years for both women and men).

For women, we found that the sequences associated with higher HIV prevalence (compared to the reference sequence) present a gap between sexual debut and first union. In particular, the sequences with the highest prevalence are those with premarital childbearing (Model 1).

For men, we found that the two sequences with a higher HIV prevalence (compared to the reference sequence, $\left( \mathrm{SU} \right)(C)$), are $\left( S \right)\left( U \right)\to(C)$ and $\left( S \right)\left( C \right)(U)$. The fact that the delayed sequence $\left( S \right)\left( U \right)\to\left( C \right)$ is significant implies that men at higher HIV risk are those who experience delayed fatherhood, rather than not experiencing it at all.

These results confirm therefore that high HIV prevalence is associated, in particular, with premarital parenthood, an event that generally leads to social stigma in sub-Saharan Africa, and that may be a signal of a disordered life style.

**Table A. Analysis limited to complete sequences for women.** Adjusted odds ratios and 95% confidence intervals for the probability of being HIV-infected, based on logistic regression models, among women, Manicaland (Zimbabwe), 2000-2011. Sample size is varying because of missing values. Only individuals with complete sequences were considered.

| **Variable** | **Category** | **Model (1)**  **Adj. OR (95% CI)** | **Model (3)**  **Adj. OR (95% CI)** |
| --- | --- | --- | --- |
| **Sequence** | | | |
|  | $\left( SU \right)(C)$ | 1.00 | 1.00 |
|  | $\left( SUC \right)$ | 1.07 (0.82,1.39) | 1.00 (0.76,1.31) |
|  | $\left( SU \right)\to(C)$ | 1.00 (0.60,1.65) | 0.99 (0.59,1.66) |
|  | $\left( S)(U \right)(C)$ | 1.42 (1.07,1.88)* | 1.18 (0.83,1.68) |
|  | $\left( S \right)\left( U \right)\to(C)$ | 1.79 (0.83,3.84) | 1.82 (0.78,4.22) |
|  | $\left( S \right)(UC)$ | 1.77 (1.22,2.56)* | 1.51 (0.99,2.29) |
|  | $\left( S \right)(C)(U)$ | 2.68 (1.39,5.13)* | 1.83 (0.82,4.04) |
|  | $\left( S \right)\left( C \right)\to(U)$ | 1.35 (0.35,5.3) | 0.76 (0.13,4.43) |
|  | $\left( SC \right)(U)$ | 2.12 (1.02,4.39)* | 1.56 (0.72,3.35) |
|  | $\left( SC \right)\to(U)$ | 5.83 (0.56,60.98) | 3.39 (0.29,39.2) |
|  | $\left( U \right)(SC)$ | 1.62 (0.62,4.21) | 1.31 (0.43,3.94) |
|  | $\left( U \right)\left( S \right)(C)$ | 0.7 (0.31,1.58) | 0.81 (0.35,1.89) |
|  | $\left( U \right)\left( S \right)\to(C)$ | 1.97 (0.35,11.03) | 1.31 (0.18,9.28) |
| **Birth cohort** | | | |
|  | *1961-1970* | 1.00 | 1.00 |
|  | *1971-1980* | 0.98 (0.71,1.35) | 0.99 (0.71,1.38) |
|  | *1981-1990* | 0.69 (0.44,1.08) | 0.73 (0.46,1.17) |
| **Setting of residence** | | | |
|  | *Rural* | 1.00 | 1.00 |
|  | *Urban* | 1.73 (1.41,2.12)* | 1.63 (1.32,2.01)* |
| **Age at interview** |  | 1.71 (1.51,1.95)* | 1.72 (1.45,2.05)* |
| **Age at interview (squared)** |  | 0.992 (0.9901,0.994)* | 0.992 (0.9902,0.994)* |
| **Age at school leaving** |  | 0.98 (0.95,1.02) | 0.98 (0.95,1.02) |
| **Age at first union** |  | 0.95 (0.89,1.02) | 0.96 (0.85,1.09) |
| **Age at first child** |  | 1.01 (0.95,1.08) | 0.9956 (0.9321,1.0635) |
| **Years since sexual debut** |  |  | 0.9919 (0.8847,1.1121) |
| **Non-regular sexual relation** | | | |
|  | *None* |  | 1.00 |
|  | *Only premarital* |  | 1.85 (1.5,2.28)* |
|  | *Only extramarital* |  | 2.25 (1.72,2.96)* |
|  | *Both* |  | 2.54 (1.5,4.31)* |
| **N** |  | 3,036 | 2,989 |

**^*^** *P-*value 0.05.

**Table B. Analysis limited to complete sequences for men.** Adjusted odds ratios and 95% confidence intervals for the probability of being HIV-infected, based on logistic regression models, among men, Manicaland (Zimbabwe), 2000-2011. Sample size is varying because of missing values. Only individuals with complete sequences were considered.

| **Variable** | **Category** | **Model (1)**  **Adj. OR (95% CI)** | **Model (3)**  **Adj. OR (95% CI)** |
| --- | --- | --- | --- |
| **Sequence** | | | |
|  | $\left( SU \right)(C)$ | 1.00 | 1.00 |
|  | $\left( SUC \right)$ | 0.71 (0.32,1.59) | 0.75 (0.33,1.67) |
|  | $\left( SU \right)\to(C)$ | 0.9 (0.39,2.05) | 0.86 (0.37,2) |
|  | $\left( S)(U \right)(C)$ | 1.29 (0.91,1.83) | 0.75 (0.48,1.16) |
|  | $\left( S \right)\left( U \right)\to(C)$ | 2.42 (1.28,4.6)* | 1.52 (0.76,3.03) |
|  | $\left( S \right)(UC)$ | 1.18 (0.75,1.84) | 0.65 (0.38,1.1) |
|  | $\left( S \right)(C)(U)$ | 1.98 (1.16,3.38)* | 1.04 (0.56,1.92) |
|  | $\left( S \right)\left( C \right)\to(U)$ | 2.54 (0.8,8.02) | 0.98 (0.29,3.37) |
|  | $\left( SC \right)(U)$ | 0.65 (0.14,3.11) | 0.47 (0.1,2.3) |
|  | $\left( SC \right)\to(U)$ | 0.69 (0.07,7.17) | 0.39 (0.04,4.3) |
|  | $\left( U \right)(SC)$ | 3.72 (0.22,62.41) | 5.49 (0.29,104.92) |
|  | $\left( U \right)\left( S \right)(C)$ | 0.73 (0.15,3.46) | 0.86 (0.18,4.19) |
|  | $\left( U \right)\left( S \right)\to(C)$ | - | - |
| **Birth cohort** | | | |
|  | *1961-1970* | 1.00 | 1.00 |
|  | *1971–1980* | 0.47 (0.33,0.67)* | 0.5 (0.35,0.72)* |
|  | *1981-1990* | 0.46 (0.25,0.83)* | 0.51 (0.28,0.95)* |
| **Setting of residence** | | | |
|  | *Rural* | 1.00 | 1.00 |
|  | *Urban* | 1.56 (1.22,2.01)* | 1.51 (1.17,1.95)* |
| **Age at interview** |  | 1.67 (1.35,2.06)* | 1.54 (1.24,1.91)* |
| **Age at interview (squared)** |  | 0.993 (0.9901,0.996)* | 0.993 (0.9904,0.996)* |
| **Age at school leaving** |  | 0.93 (0.89,0.96)* | 0.93 (0.89,0.97)* |
| **Age at first union** |  | 1.03 (0.97,1.09) | 1.08 (1.01,1.16)* |
| **Age at first child** |  | 0.99 (0.93,1.05) | 0.98 (0.92,1.04) |
| **Years since sexual debut** |  |  | 1.07 (1.02,1.12)* |
| **Non-regular sexual relation** | | | |
|  | *None* |  | 1.00 |
|  | *Only premarital* |  | 1.56 (1.01,2.41)* |
|  | *Only extramarital* |  | 1.52 (0.84,2.74) |
|  | *Both* |  | 2.37 (1.47,3.8)* |
| **N** |  | 1,937 | 1,918 |

**^*^** *P-*value 0.05.
